# Subacute Cardiac Tamponade With Massive Pericardial Effusion

**DOI:** 10.7759/cureus.32250

**Published:** 2022-12-06

**Authors:** Aung Hein, Yin H Wai

**Affiliations:** 1 Department of Cardiology, University Hospitals Dorset NHS Foundation Trust, Bournemouth, GBR; 2 Department of Respiratory Medicine, Poole Hospital, Poole, GBR

**Keywords:** cardiac tamponade, emergency echocardiography, massive pericardial effusion, haemodynamic compromise, pericarditis, tamponade

## Abstract

Subacute cardiac tamponade is a diagnostic challenge for clinicians because the symptoms would be non-specific upon presentation. The onset of cardiac tamponade may vary depending on the rate of accumulation and compensatory mechanism of the fibroelastic pericardial sac. In the case of subacute tamponade with effusion without cardiac arrest, it is usually challenging for the clinician to make the decision for urgent drainage. Usually, cardiac tamponade is treated as a medical emergency, and it occurs when fluid accumulated in the pericardial sac compresses the heart causing haemodynamic compromise and cardiac arrest. In our case, a 40-year-old man presented with a seven-day history of significant shortness of breath. He presented to the emergency department and the chest X-ray showed a large cardiac silhouette, which suggested a large pericardial effusion. ECG revealed minor changes in the heights of QRS complexes. Point-of-care echocardiography showed a large pericardial effusion, and he was immediately admitted to the cardiac unit. Urgent departmental echocardiography confirmed massive pericardial effusion with features of subacute tamponade. The patient was sent to the cardiac catheterisation lab and a total of approximately 4.2 litres of pericardial effusion was drained, while he was closely monitored for the risk of rapid physiologic decompensation after drainage. Pericardial fluid culture did not show any evidence of microorganism growth. The connective tissue disease screen was negative. CT scan did not show any stigmata of occult malignancy or features of infection. The coronavirus disease 2019 (COVID-19) polymerase chain reaction test was negative. He had rapid symptomatic improvement after the effusion was drained and recovery was uneventful. He was discharged from the hospital with a follow-up plan. We concluded that it was a case of subacute cardiac tamponade due to a massive pericardial effusion of idiopathic or subclinical viral causes. Clinical presentation of subacute cardiac tamponade could be easily missed, and a detailed assessment of the effusion with echocardiography was very helpful in making decisions for the management.

## Introduction

Cardiac tamponade is a condition when the heart is compressed by accumulated fluid with increased intrapericardial pressure causing haemodynamic instability. It could either be acute or subacute depending on the cause of fluid accumulation inside pericardial layers. The pericardial sac is a double-layered fibroelastic sac that may contain serous fluid of up to 50 ml in normal individuals. Accumulation of fluid could be secondary to idiopathic pericarditis, infective pericarditis, connective tissue diseases, neoplastic, iatrogenic causes such as cardiac procedures, aortic dissection, diseases with fluid overload such as renal failure, post-myocardial infarction, and amyloidosis [1]. With the increased amount of pericardial fluid and rising intrapericardial pressure, when compensatory elasticity in relation to the maximal volume capacity of the pericardium is exceeded, cardiac chambers become small due to compression by the fluid. Reduction in cardiac chamber sizes impairs cardiac filling, low venous return, and poor cardiac output [2]. In cardiac tamponade, the timing of presentation would be acute or subacute, and the latter symptoms may appear from days to weeks. Common signs and symptoms in acute tamponade include chest pain, shortness of breath, tachypnoea, tachycardia, hypotension, elevated jugular venous pressure, pericardial rub, muffled heart sound, and pulsus paradoxus. In subacute tamponade, presentations would be symptoms such as fatigue, progressive breathlessness, leg swellings, and tiredness. Physical findings in subacute tamponade share similar features with acute tamponade and it is described as a classical Beck's triad, which includes hypotension, high jugular venous pressure, and muffled heart sound. We present a case of massive pericardial effusion with relatively stable haemodynamic parameters but unstable features on echocardiography.

## Case presentation

**Figure 1:**
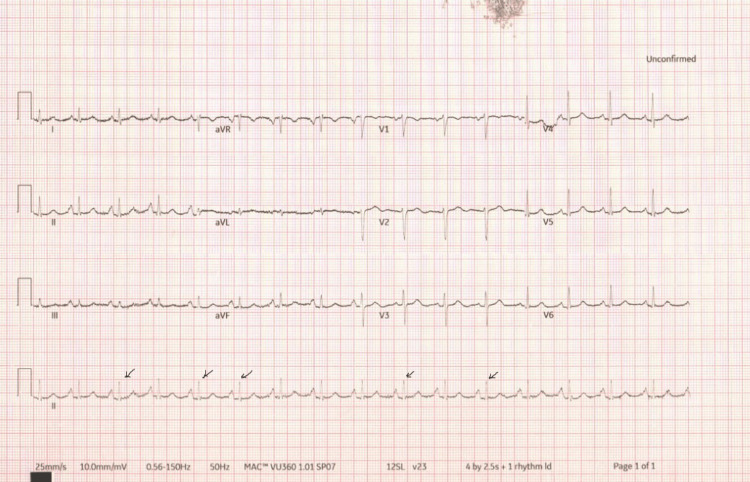
ECG ECG with subtle changes in QRS complexes.

**Figure 2:**
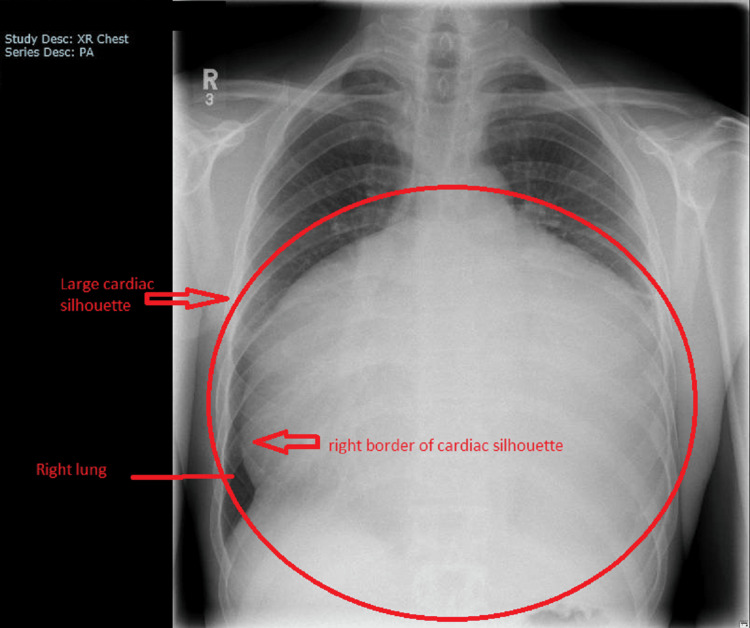
Chest X-ray Chest X-ray showing large cardiac silhouette suggestive of massive pericardial effusion

A 40-year-old male patient presented to the emergency department with a seven-day history of severe shortness of breath on exertion. He was usually very fit and well. He had significant shortness of breath on exertion upon presentation. He had a mild dry cough but denied other symptoms such as fever, chest pain, abdominal pain, and genitourinary symptoms. He had a past medical history of mild asthma for which he was on inhaled salbutamol and he rarely needed to use the inhaler. He was not on any regular medication. He was an active smoker with 30 packs per year history. On examination, he was breathless, had muffled heart sounds, and there were no leg swellings. He had bilateral expiratory wheezes on auscultation. The rest of the systemic examinations were unremarkable. His observations were stable with a blood pressure of 140/100 mmHg, heart rate of 96 bpm, respiratory rate of 23 per minute, oxygen saturation of 96% on air, and temperature of 36.4°C. His first ECG showed sinus rhythm with a ventricular rate of 96 bpm, and there were subtle variations in QRS complexes (Figure 1). His chest radiograph showed a large cardiac shadow with clear lung fields (Figure 2). His initial blood tests were unremarkable with mild fluctuations of inflammatory markers in subsequent blood tests (Table 1).

**Table 1:** Haematology, troponin T, coagulation profile with D-dimer, C-reactive protein, renal function test, liver function test, and calcium levels APTT, activated partial thromboplastin time; GFR, glomerular filtration rate; MDRD, modification of diet in renal disease; ALT, alanine aminotransferase.

	Day 1	Day 2	Day 3	Day 4	Day 5	Day 6	Day 7	Day 8	Day 9	Reference range	Units
Haematocrit	0.47	0.42	0.45	0.51	0.51	0.46	0.48	0.49	0.48	0.4-0.5	L/L
Lymphocyte count	1.3	1.3	1.1	0.8	1.1	0.8	1	1.1	1.3	1.0-3.0	10*9/L
Neutrophil count	6.5	5.3	7.7	10.4	10.3	8.6	6.1	5.1	4.9	2.0-7.0	10*9/L
Haemoglobin estimation	155	142	151	170	168	153	159	161	158	130-170	g/L
Platelet count	143	119	147	159	177	194	235	293	325	150-410	10*9/L
Mean corpuscular volume (MCV)	96	94	95	95	94	95	96	94	94	83-101	fL
Total white cell count	9.7	9.1	10.8	14	14.7	13.4	10.8	9.9	10.5	4.0-10.0	10*9/L
APTT ratio	1.1									0.8-1.2	
Fibrinogen level	3.09									1.88-4.15	g/L
International normalised ratio	1									0.9-1.2	
Prothrombin time	13.8									11.6-14.6	Seconds
Serum C-reactive protein level	1.4	1.4		1	29		4.5	29	13	0-9	ng/mL
D-dimer level	261									<500	ng/mL
Serum troponin T level	13									<14	ng/L
Serum creatinine	61	67	66	52	56	51	88	57	55	59-104	µmol/L
GFR calculated abbreviated MDRD	90	90	90	90	90	90	90	90	90	-	mL/min/1.73m*2
Serum potassium	4.4	3.7	4	4.6	5.1			5.1	4.4	3.5-5.0	mmol/L
Serum sodium	140	140	140	135	139	136	138	136	136	132-146	mmol/L
Serum urea level	2.6	3.6	3.7	3.4	3.6	2.7	14	3.9	4.4	2.5-6.7	mmol/L
Serum albumin	45									35-48	g/L
Serum alkaline phosphatase	100									30-100	U/L
Serum ALT level	42									0-35	µmol/L
Serum total bilirubin level	13									0-17	µmol/L
Serum calcium	2.39									2.20-2.60	mmol/L
Corrected serum calcium level	2.39									2.2-2.6	mmol/L

We did urgent point-of-care echocardiography, which showed that the patient had a massive pericardial effusion and a swinging heart. The cardiology team was urgently notified and discussed and the patient was admitted to the coronary care unit. The departmental echocardiography was performed in the coronary care unit, which showed echocardiographic features of cardiac tamponade.

In transthoracic echocardiography, there are several useful features in assessing pericardial effusion. These include measurement of pericardial effusion, assessing chambers, particularly the right atrium and ventricle, size of inferior vena cava (IVC), interventricular septal “bounce”, and assessment with Doppler for haemodynamic changes.

**Figure 3:**
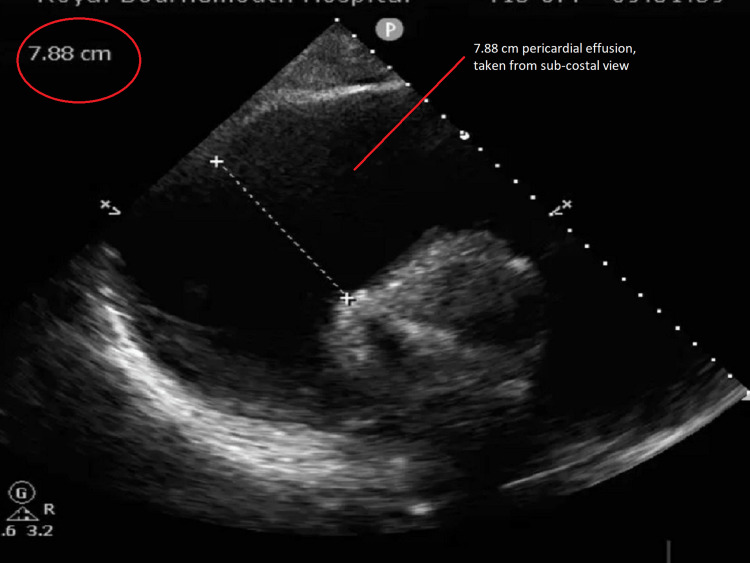
Transthoracic echo (subcostal view) Massive pericardial effusion measured up to 7.88 cm.

In terms of quantitative measurement, the patient had a large circumferential pericardial effusion and from the subcostal view, it was 7.88 cm (Figure 3). The severity of pericardial effusion on echocardiography is usually classified into mild, moderate, and severe, as per the measurement. Effusion size below 1 cm is mild, 1-2 cm is moderate, and more than 2 cm is severe. In our case, the effusion size was between 5 and 7.88 cm and it was a severe pericardial effusion.

As for another parameter, right atrial collapse is accepted as a sensitive feature of cardiac tamponade, and it occurs in the relaxation phase when the pressure inside the chamber is low in comparison with pericardial fluid pressure [3]. In our case, we can see right atrial collapse also known as inversion of the right atrium seen on the apical four-chamber view (00:13, Video 1). During the relaxation phase, i.e., in diastole, right ventricular pressure would also be low and right ventricular collapse could also be seen in the case of tamponade [4]. In our case, we saw the right ventricular collapse in the parasternal long-axis view (00:04, Video 1).

**Video 1:** Massive pericardial effusion with subacute tamponade Massive pericardial effusion and swinging heart.

**Figure 4:**
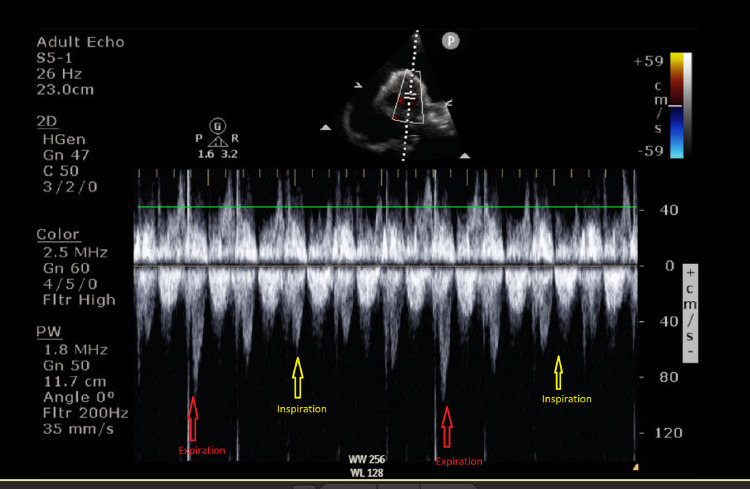
Transthoracic echo Variability with respiration at the left ventricular outflow tract (pulsed-wave Doppler).

**Figure 5:**
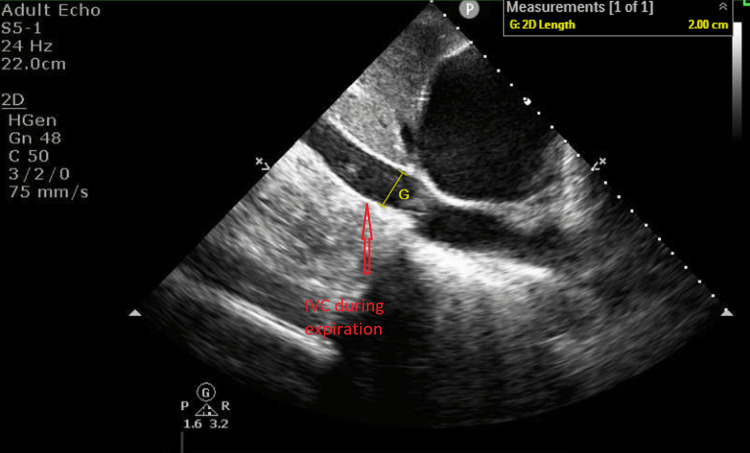
Transthoracic echocardiography (subcostal view) The size of the inferior vena cava was 2.0 cm during expiration. IVC, inferior vena cava.

Another echocardiographic parameter is the dilated IVC and its normal range is 2.1 +/- 0.6 cm [5]. In our case, his IVC size was 2.0 cm and it was in the normal range. There is another important echocardiographic feature of IVC, which is its collapsibility [6]. In normal circumstances, the IVC collapses in response to inspiration and the collapsibility is more than 50%. In our case, the IVC size during inspiration during expiration was 2.0 cm and inspiration was 1.57 cm and its collapsibility was less than 50% during inspiration, which meant his collapsibility was poor, supporting cardiac tamponade (Figures 4, 5).

There is another non-specific feature for the diagnosis of cardiac tamponade, which is called the septal bounce. It is the interventricular septum moving towards the left ventricle initially and then moving away in early diastole. It is a phenomenon accentuated by inspiration and reversed by expiration. This feature is seen in situations such as pulmonary embolism, pericardial disease, and cardiac tamponade [7]. In this case, we looked at the septum, and it looked like there was a septal bounce. However, due to the rapidly swinging heart, it was inconclusive (Video 1).

In the case of tamponade, pulsed-wave Doppler is a very useful tool to assess. In normal respiration, spontaneous venous return to the right heart increases in relation to intrathoracic pressure gradient changes and increased filling pressure to the right ventricle limits left ventricular end-diastolic volume and pressure [8]. The right and left ventricles interact with each other and the expansion of one chamber could compress the other. This is known as ventricular interdependence and the exaggerated phenomenon is seen as a septal bounce, as mentioned in this article [9,10].

**Figure 6:**
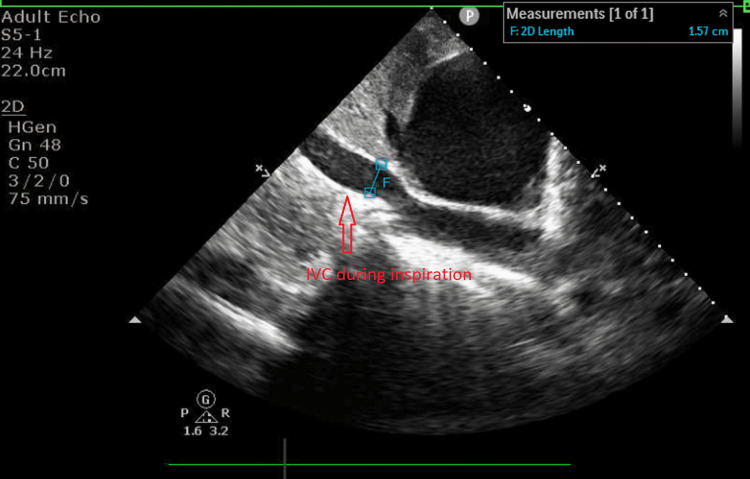
Transthoracic echocardiography (subcostal view) The size of the inferior vena cava was 1.57 cm during inspiration. IVC, inferior vena cava.

Doppler echocardiography can provide very useful haemodynamic assessment in multiple situations. In the case of tamponade, pulsed-wave Doppler is a very useful tool to assess by exploiting the physiology we already understood. We generally can assess flow across the tricuspid valve, mitral valve, right ventricular outflow tract, and left ventricular outflow tract. In cardiac tamponade, transmitral flow assessment shows a 25% reduction of peak E wave velocity in inspiration. For the tricuspid valve, peak E wave velocity will drop 40% during expiration. In normal circumstances, the variation of the peak E wave in the mitral valve is less than 15% and for the tricuspid valve, it is less than 25% [11]. On the right and left ventricular outflow tracts in normal patients, the variations of peak velocity and velocity time integral in relation to the respiratory cycle are usually less than 10% in pulsed-wave Doppler assessment [12]. In the case of cardiac tamponade, there is a significant variation in these parameters. During inspiration, at the left ventricular outflow tract, a decline in peak velocity can be seen and it is more than 10%. However, during inspiration, pulsed-wave Doppler measurement at the right ventricular outflow tract will increase by more than 10%. In our case, we can see obvious differences in peak velocities. It was measured at the left ventricular outflow tract and the decline in peak velocity is more than 10% in our patient. It indicated that the patient was in a state of tamponade. As discussed in this article, cardiac output decreases during inspiration. In tamponade, we see an exaggerated response of this physiologic process and in our case, we have recorded this phenomenon (Figure 6). This is the echocardiographic equivalence of pulsus paradoxus [13,14].

The size of the pericardial effusion, the IVC collapsibility, the appearance of chambers, and the drop of peak velocities in pulsed-wave Doppler velocities at the left ventricular outflow tract supported the fact that the patient was in the state of cardiac tamponade.

With stable blood pressure, there was a question of whether the patient needed an urgent drain overnight. In the literature, there were reports that patients with subacute cardiac tamponade may not present with low blood pressure [15,16]. Additionally, he was very symptomatic with significant shortness on exertion and echocardiography parameters were suggestive of cardiac tamponade. Therefore, we followed the triage cardiac tamponade pathway proposed by the European Society of Cardiology. The patient had dyspnoea, large cardiac shadow on chest X-ray, echocardiographic pulsus paradoxus, effusion size more than 2 cm, swinging heart, and collapsing chambers, which indicated urgent pericardiocentesis after excluding contraindication. Therefore, we decided to continue the plan of urgent pericardiocentesis.

**Figure 7:**
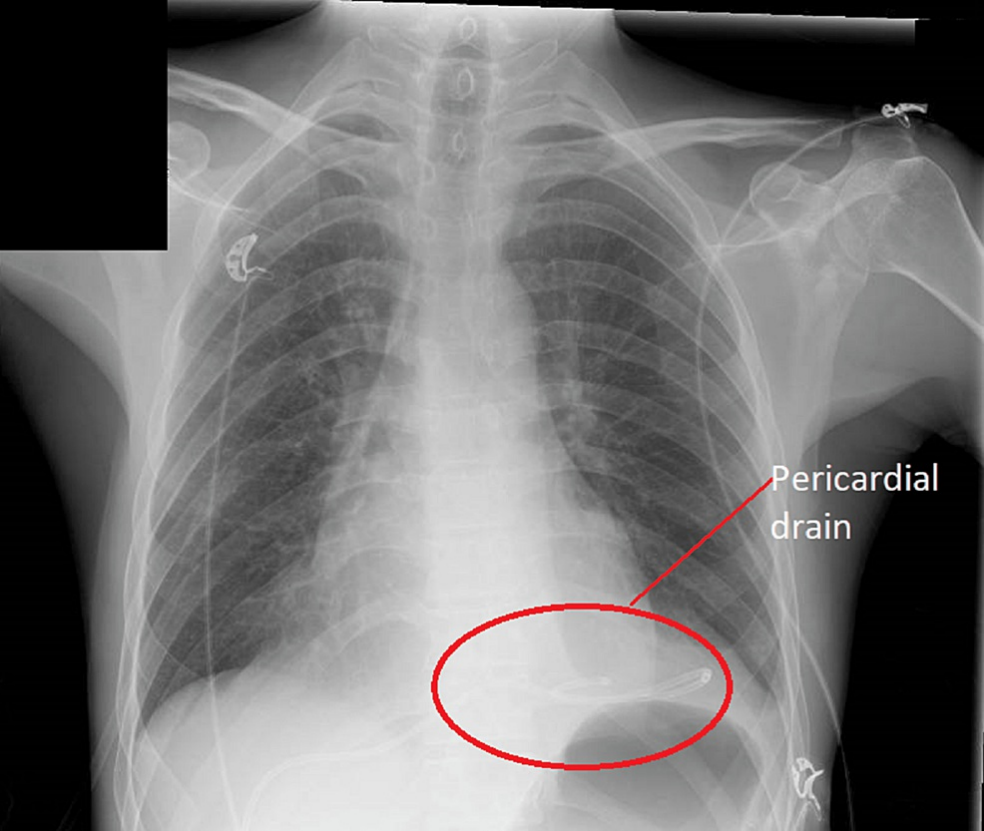
Chest X-ray Significant improvement in cardiac shadow after draining. Pericardial drain in situ.

The patient was then sent to the cardiac catheterisation laboratory urgently and had a pericardial drain inserted under fluoroscopy guidance. Macroscopically, it was of straw colour. A fluid sample was sent for culture. It was drained serially for 500 ml every four to six hours due to the risk of rapid haemodynamic decompensation. A total of 4276 ml was drained. The microbiology result showed that it was negative for bacterial and mycobacterial cultures. Chest X-ray appearance improved significantly after we drained the pericardial effusion (Figure 7).

**Figure 8:**
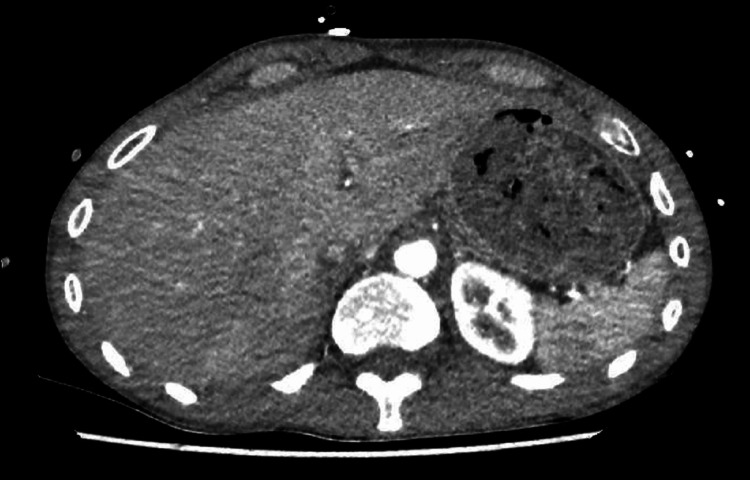
CT of the chest, abdomen, and pelvis No evidence of occult malignancy was seen.

**Figure 9:**
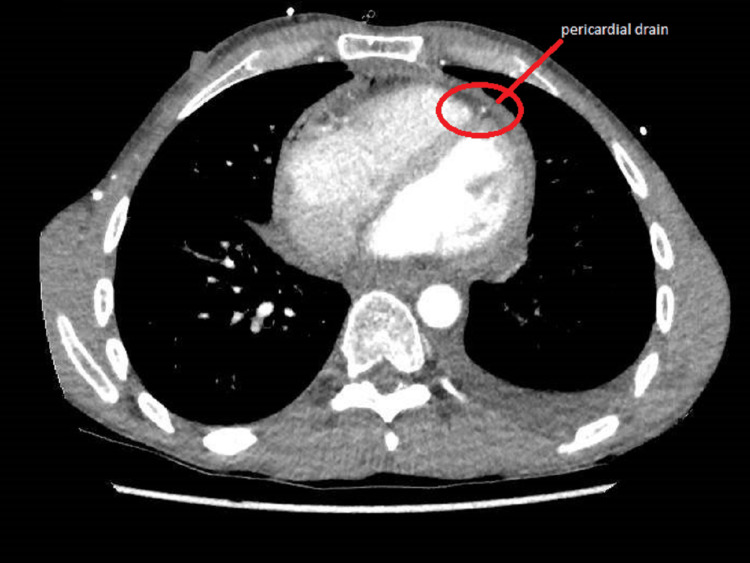
CT of the chest, abdomen, and pelvis Minimal residual pericardial fluid with the pericardial drain in situ.

For non-infective aetiology of pericarditis causing pericardial effusion, we sent an auto-immune screen. The auto-immune screen included anti-double stranded DNA antibody, anti-Smith antibody, antibody to proliferating cell nuclear antigen (PNCA), antibody to ribosomal P (Rib-P), antibody to U1 spliceosome RNA (U1-snRNP), anti-Ro and anti-La antibodies, anti-topoisomerase I antibody (anti-Scl-70), anti-centromere antibody (CENP), anti-fibrillarin antibodies, anti-RNA polymerase III antibody, anti-Jo1 antibody, anti-Mi2 antibody, and anti-PM/Scl antibody. The screen was negative. Myeloperoxidase and proteinase 3 antibodies were also negative. We looked for other causes that might have contributed to pericardial effusion by performing CT of the chest, abdomen, and pelvis. CT scan did not reveal any evidence of occult infections or malignancy (Figure 8) and showed minimal residual effusion with a pericardial drain in situ (Figure 9).

Serial SARS-CoV-2 RNA (coronavirus disease 2019 polymerase chain reaction) tests were negative. After 48 hours of drainage, we removed the drain without complications. His symptoms improved dramatically after we drained the pericardial effusion. We treated him as subacute cardiac tamponade with massive pericardial effusion. He was discharged after staying in the hospital for nine days with a follow-up plan.

## Discussion

Cardiac tamponade is generally treated as a medical emergency and a well-listed cause for cardiac arrest in advanced life support guidelines [17]. In acute settings with uncompensated myocardium, rapid accumulation of fluid may lead to cardiac tamponade. Such a presentation would be associated with other obvious pathologies such as chest pain with acute aortic dissection or chest trauma [18,19]. However, in subacute settings, symptoms of the pericardial effusion may range from no symptoms or generalised fatigue to tamponade complicated with cardiac arrest. In subacute or chronic pericardial effusion, the pericardial sac would compensate with its elasticity until it reaches maximal tolerance [20]. With the growing volume of pericardial effusion, the fluid will compress cardiac chambers and affect ventricular filling. High intrapericardial pressure will reduce venous return, which will lower cardiac output. As a compensatory mechanism, the patient may have tachycardia and tachypnoea. Due to poor cardiac output, there would be narrow pulse pressure. Typical symptoms would include shortness of breath, fatigue, and leg swelling. While acute tamponade usually presents with rapid decline, there would be a thin line between large stable pericardial effusion and subacute tamponade, and it is a diagnostic challenge [21].

In our case, a relatively young patient had excessive shortness of breath on exertion over seven days and it was quite unusual for him. His haemodynamic parameters were not alarming on the first encounter. Once we had done a chest X-ray, it provided an excellent clue for his symptoms. At first glance, it looked like it was an unremarkable X-ray of a young male. When we followed the right border of the cardiac silhouette, it clearly showed an impressive cardiac silhouette. We did not see the left costophrenic angle of the chest X-ray. Following the chest X-ray, ECG provided some useful information. When we carefully looked at QRS complexes, there were subtle changes in the heights of QRS complexes. When there was a strong suspicion of a large pericardial effusion, we did point-of-care echocardiography and it showed a massive pericardial effusion. The heart was swinging in the pericardial fluid and the cardiac chambers looked small. These were clear indications to get the cardiology team involved.

Clinically, the patient's observations including blood pressure, heart rate, respiratory rate, and saturation were stable. After departmental echocardiography was done, the scan confirmed massive pericardial effusion with echocardiographic features of cardiac tamponade. On the scan, the effusion was obviously a very large effusion. The chambers were small and visibly collapsing. Due to the size of the effusion, it was very difficult to assess flow across the mitral valve and tricuspid valve. The pulsed-wave Doppler peak velocities at the left ventricular outflow tract showed a drop in inspirations and this feature was accepted as echocardiographic equivalence to pulsus paradoxus [22]. The stable clinical observations were falsely reassuring, and it is not uncommon that decision-making was difficult [21,23]. However, in the literature, there were cases reported with cardiac tamponade without hypotension [24].

For symptomatology, looking back at his baseline functional status, the patient was obviously a very fit and well man without any co-morbidity limiting his quality of life. Since he started having the symptoms, shortness of breath was quite debilitating and unusual for him. We suspected that he was in the state of subacute cardiac tamponade due to a large pericardial effusion. After we performed point-of-care and departmental echocardiography, we were convinced that he was in cardiac tamponade. With the cardiology team's involvement, the patient was sent to the cardiac catheterisation laboratory and pericardial fluid was drained. If clinicians delayed intervention due to stable blood pressure, there was a risk of cardiac arrest due to tamponade. In hospitals, we see patients with pericardial effusion often and it was not an uncommon finding. However, it was very important to identify signs of pericardial tamponade clinically and on echocardiography. Considering all factors from symptoms to echocardiography is important. In our patient, the fluid was serially drained due to the risk of developing pericardial decompression syndrome and we drained nearly 4.2 L of fluid [25].

To understand the underlying aetiology of pericardial effusion, we sent pericardial fluid samples for culture and sensitivity including mycobacterial studies. The fluid sample was negative for acid-fast bacilli and the mycobacterial culture was negative as well. Culture and sensitivity for other bacterial studies were also negative. Throughout the hospital stay, we involved local microbiologists in regular discussions. With negative cultures, we considered viral causes as possible aetiology, and we requested opinions from microbiologists. We agreed that it did not change the management plan and we did not do virology studies from the fluid samples. Since we often saw pericardial effusion in cases with underlying malignancy, we did a CT scan of the chest, abdomen, and pelvis, which did not show any occult pathology such as a malignancy or infection. After we had ruled out occult malignancy and infections on the CT, we sent an auto-immune/connective tissue disease screen to rule out auto-immune causes. When all results were normal, we concluded that the patient might have had idiopathic pericarditis or subclinical viral pericarditis with massive pericardial effusion.

The patient had rapid symptomatic improvement after the pericardial fluid was drained. Upon discharge with a follow-up plan, he had a complete recovery from the symptoms he suffered.

## Conclusions

Subacute cardiac tamponade may present with non-specific symptoms such as breathlessness alone or fatigue and it is vitally important to get the correct diagnosis in time. Pericardial effusion would be asymptomatic in many cases, but it may lead to subacute cardiac tamponade if the fluid accumulated becomes large enough to compress cardiac chambers. Traditionally, blood pressure, heart rate, and respiratory rate have been regarded as good indicators of cardiac tamponade, and in our case, the patient's strong physical status would have insulated him from rapid physiologic decompensation and this case would be one of the examples that parameters such as blood pressure and heart rate would be normal. We have learned from the case that detailed history and accurate symptom analysis are very important in cases with vague presentations. Point-of-care echocardiography is non-invasive and could provide very useful information for emergency clinicians and specialists. Furthermore, detailed assessments from effusion, chamber, and haemodynamic status should be done with echocardiography since subacute cardiac tamponade would be diagnostically challenging and a cause of difficult management decisions.
